# Validation of Task-Specific Rating Scale for Open Balloon Catheter Arterial Embolectomy: An Assessor-Blinded Quasi-Experimental Pilot Study

**DOI:** 10.3400/avd.oa.22-00047

**Published:** 2022-12-25

**Authors:** Nadeem Ahmed Siddiqui, Shiraz Hashmi, Iram Naz, Ziad Sophie

**Affiliations:** 1 Section of Vascular Surgery, Aga Khan University Hospital, Karachi, Pakistan; 2 Department of Surgery, Aga Khan University Hospital, Karachi, Pakistan

**Keywords:** embolectomy, task-specific rating scale, validation, vascular surgery training, Global Rating scale

## Abstract

**Objective:** To develop and validate a task-specific rating scale (TSRS) by comparing with the Global Rating Scale (GRS) for the evaluation of brachial artery embolectomy (BAE).

**Methods:** Participants were divided into expert and novice groups who were oriented on the locally developed simulator model. The following day, an embolectomy procedure was performed independently by the participants and graded by two independent assessors using the GRS and TSRS. Validity was evaluated using Pearson’s correlation coefficient (r), reliability by the interclass correlation coefficient (ICC), and agreement by Bland–Altman plots. A p-value <0.05 was considered significant.

**Results:** Thirty-two participants were enrolled in this study. The overall TSRS was found to be a valid assessment tool (r=0.82; 95% confidence interval [CI]: 0.66, 0.91; p<0.001). Domain-specific analyses showed a moderate positive association between all domains (p<0.05), except for instrument handling (r=0.09; 95%CI: −0.27, 0.42; p=0.642). The ICC for overall scores showed excellent reliability for both instruments, GRS and TSRS, with values of 0.97 and 0.92, respectively.

**Conclusion:** The TSRS was found to be a valid and reliable assessment tool for BAE; however, for some domains, such as instrument handling and time and motion, it has limited reliability.

## Introduction

The technical ability of a clinician is one of the most important components of surgical competency. There is an unquestionable need to standardize the evaluation process of residents and fellows during training to ensure robust measurements of efficiency and competence.^1–3)^ It has been found that the subjective assessment of technical skills through direct task observation in the operating room, without a structured objective scale, has poor interobserver reliability.^1)^ Various surgical evaluation instruments have been developed to achieve this goal. Ample literature now exists recommending the use of the Global Rating Scale (GRS), which could be used to assess a trainee’s surgical skills on agreed-upon criteria, to provide an objective and reproducible assessment.^1, 3, 4)^ However, the GRS is generic and lacks the ability to assess the specific aspects of diverse surgical procedures performed in various subspecialties,^2)^ particularly vascular procedures. Although many procedure-specific checklists and the Objective Structured Assessment of Technical Skills (OSATS) have been developed to evaluate vascular surgery procedures, most of them are either in the process of validation or not yet validated. Combining the GRS and procedure-specific checklists by including additional safety and efficacy items has been found to be a more effective and reliable assessment for surgical dexterity.^5)^

Vascular surgery is a technically demanding specialty where the scrupulous assessment of technical skills is very important. Brachial artery embolectomy (BAE) is an emergency procedure, and patients often present in the middle of the night to the emergency room, providing few opportunities to train and assess residents and fellows involved in care. Although the incidence of upper limb amputation after BAE is low,^6)^ it is associated with significant emotional, social, and financial consequences.^7)^ Therefore, optimal assessment and credentialing are of vital importance to enable residents, fellows, and junior faculty to perform embolectomies safely and independently.

The integration of a validated procedure-specific evaluation checklist can help in the more uniform and reproducible assessment of technical and cognitive skills of trainees. The Intercollegiate Surgical Curriculum Program (ISCP) has developed some procedure-based assessment checklists, including embolectomy,^8)^ but these are not validated. Considering the lack of availability of a validated checklist for BAE, we aimed to develop and validate a procedure-specific assessment checklist for balloon catheter BAE for surgical trainees. The primary objective of this study was to develop and validate a task-specific rating scale (TSRS) by comparing the TSRS with the GRS to evaluate the procedural steps of BAE. The secondary objective was to estimate criterion cut-off points of the TSRS against overall GRS binary scores to declare trainees as successful candidates.

## Materials and Methods

An assessor-blinded quasi-experimental study conducted at the Section of Vascular Surgery, Department of Surgery, Aga Khan University Hospital, Karachi. Ethical approval was obtained from the institutional ethical review committee before initiation of the study.

### Development and finalization of assessment tools

Two assessment tools were concurrently used to assess the technical competency of trainees in performing BAE.

1. A TSRS was developed using the Delphi technique by a group comprising three senior vascular surgeons (content experts IN, ZS, ZR), one research specialist (SH), and two medical educationists (QR, AS). The Textbook of Vascular Surgery^9)^ was also used. During focus group discussions, the group used the procedure-based assessment checklist developed by the ISCP to develop the TSRS. The group also used GRS domains as a reference (**Appendix 1**) and added items to make a comprehensive assessment of the trainee, ensuring the correct steps of the procedure in the correct sequence in the new TSRS checklist. The seven GRS domains were further expanded in an itemized fashion, ensuring that all key steps of BAE have been captured; thus, a 26-item TSRS checklist was generated for this procedure (**Appendix 2**). Like the GRS, completion of the task and efficiency of each item were assessed on a 5-point Likert scale, making a total of (26×5) 130 scores (**Appendix 1**).

2. The GRS is a seven-item validated tool based on the OSATS to evaluate the performance of surgical skills used concurrently with the TSRS checklist to assess the technical skills of the study participants,^3, 10)^ with a total of (7×5) 35 scores (**Appendix 2**).

### Simulator

**Figure 1:**
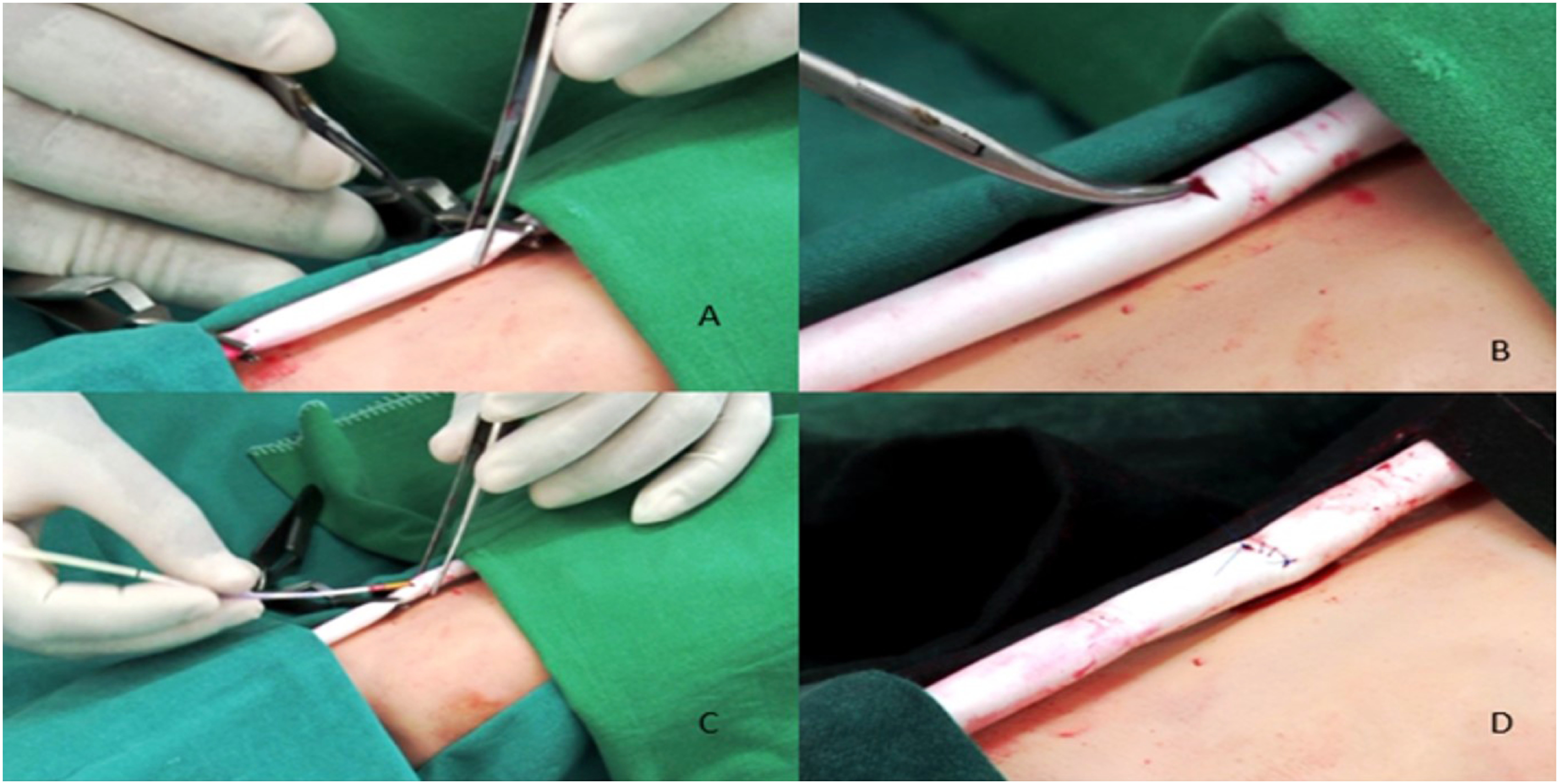
Fig. 1 A locally developed simulation model of brachial artery embolectomy. (**A**) arteriotomy, (**B**) thrombectomy, (**C**) balloon catheter embolectomy, (**D**) closure of arteriotomy.

A locally developed simulator with a rubber arm (lab model; made in the USA), plastic tubes, 6-mm polytetrafluoroethylene grafts that are 8 cm long, artificial blood (normal saline with red color), and clots (made of cellulose) were used to teach and assess the arterial embolectomy procedure (**Fig. 1**). This cost-effective simulator was tested before the workshop to ensure its smooth and efficient functioning. The approximate cost for the development of this simulator was about USD 50. The model was assembled and reassembled with new clotting material and artificial blood was filled and refilled before the beginning of each procedure.

### Assessor training

A week before the planned date of the workshop, a 1-day orientation session for the assessors was conducted on both GRS and TSRS tools by the primary investigators. The assessors (ZS, AB) had at least 10 years of experience of independent practice in vascular surgery. The assessors were educated about the use of GRS and TSRS as evaluation tools. Orientation of the video recording and the role of surgical assistants were ensured and emphasized during these sessions. All assessors were requested to rate at least four pre-recorded procedural videos to assess inter-rater reliability, which fell within an excellent range (interclass correlation coefficient [ICC] >85%).

### Participants

Senior residents from general surgery (year three onwards) and fellows of vascular surgery were invited to participate in the workshop (novice group). Vascular surgeons certified by the national/international board and who had at least 3 years of experience performing independent arterial embolectomy (expert group) were also invited.

### Assessment technique

Informed consent was obtained from each participant to enroll in the study. On day 1, an orientation video of the simulation model and steps of arterial embolectomy were presented to all participants via an interactive session. Participants were then allowed to practice the procedure on the simulator. During these drills, formative feedback was provided to the participants by the assigned group facilitators. During this session, each participant from both groups was given adequate time (1 h) to practice and become familiar with the simulator. On day 2, participants were asked to perform an embolectomy independently on the same model with the help of an assistant, who was a trained vascular surgery technician, who was instructed to help only when asked by participant. Twenty minutes were assigned to each participant to accomplish the given task. Blinded video recording was performed for each trainee while performing the procedure. The performance of trainees was graded at a later stage by two independent assessors concomitantly using the GRS and TSRS on the pre-recorded videos. Assessors were kept blinded by assigning unique identity codes on participants’ gloves so that they were easily visible to the assessors without recognizing them.

### Statistical analyses

Data were analyzed by using the Statistical Package for the Social Sciences (version 22.00) and Medcalc software (version 19.2). The normality of continuous variable was assessed by the Shapiro–Wilk test. The average of the TSRS scores for each corresponding domain of the GRS was computed and compared. Pearson’s correlation coefficients (r) with 95% confidence intervals (CIs) were computed to assess the concurrent validity. Inter-rater reliability was assessed by the ICC and 95%CI and agreement in terms of percent difference by Bland–Altman plots between the two instruments.^11)^ Receiver operating curve (ROC) analyses were conducted to estimate criterion cut-off points of the TSRS against overall GRS binary scores of 65% to declare trainees as successful candidates. Mean differences of experts and novices were measured by using the Student t-test or Mann–Whitney U test and the chi-squared test or Fisher’s exact test, as appropriate. A p-value of less than 0.05 was considered statistically significant.

## Results

A total of 32 participants, 22 novices and 10 experts, successfully completed the workshop. The novice group consisted of three vascular surgery fellows and 19 senior general surgery residents. The maximum scores achieved on the GRS and TSRS were 23.92±6.02 and 116.27±6.34, respectively. Pearson’s correlation coefficients (r) and 95%CIs of overall and domain-specific estimates are presented in **Table 1**. There was a strong correlation between the overall GRS and mean TSRS score (r=0.82; 95%CI: 0.66, 0.91; p<0.001). Domain-specific analyses showed a moderate association range (r=0.43–0.60; p<0.05), except for instrument handling that showed a weak association (r=0.09; 95%CI: −0.27, 0.42). The GRS reliability coefficient for overall and domain-specific scores were in excellent ranges (ICC: 0.81–0.97; **Table 2**). The TSRS reliability coefficients also fell in excellent ranges (ICC: 0.71–0.97), except for time and motion and instrument handling (ICC: 0.23; 95%CI: −0.57, 0.63; and 0.38; 95%CI: −0.27, 0.70, respectively). Bland–Altman plots showed an overestimation in the tissue and quality of product domains and underestimations in all other domains, including overall scores. Assuming an acceptable difference of <15%, overall and all domains fell under acceptable ranges, except for knotting and suturing, which was above 19%.

**Table 1:** Table 1 Mean Global Rating Scale (GRS) and Task-Specific Rating Scale (TSRS) scores and construct validity of both instruments assessed by Pearson’s correlation coefficient and 95%CI, by task; n=32

Domains	Mean GRS ±SD	Mean* TSRS ±SD	r^†^ (95%CI)	p-Value
Respect for tissue	3.5±0.81	3.96±0.36	0.49 (0.18, 0.72)	0.004
Time and motion	3.47±1.03	4.34±0.25	0.43 (0.10, 0.68)	0.014
Instrument handling	3.44±1.05	4.67±0.23	0.09 (−0.27, 0.42)	0.642
Knotting and suturing	3.19±1.20	4.64±0.34	0.58 (0.28, 0.77)	0.001
Use of assistant	3.19±1.12	4.36±0.67	0.59 (0.31, 0.78)	<0.001
Procedural flow	3.64±0.92	4.69±0.27	0.45 (0.12, 0.69)	0.009
Quality of final product	3.5±1.00	4.35±0.60	0.60 (0.32, 0.79)	<0.001
Overall	23.92±6.02	31.0±1.83	0.82 (0.66, 0.91)	<0.001

* Mean scores of theme-specific items. ^†^ r=Pearson’s correlation coefficients.CI: confidence intervals; SD: standard deviation

**Table 2:** Table 2 Reliability of the Global Rating Scale (GRS) and Task-Specific Rating Scale (TSRS); n=32

Task	Interclass correlation coefficients* (95%CI)			
	GRS	p-Value	TSRS	p-Value
Respect for tissue	0.93 (0.86, 0.97)	<0.001	0.71 (0.42, 0.86)	<0.001
Time and motion	0.94 (0.88, 0.97)	<0.001	0.23 (−0.57, 0.63)	0.233
Instrument handling	0.92 (0.84, 0.96)	<0.001	0.38 (−0.27, 0.70)	0.095
Knotting and suturing	0.92 (0.83, 0.96)	<0.001	0.94 (0.87, 0.97)	<0.001
Use of assistant	0.93 (0.85, 0.96)	<0.001	0.85 (0.70, 0.93)	<0.001
Procedural flow	0.81 (0.62, 0.91)	<0.001	0.91 (0.82, 0.96)	<0.001
Quality of final product	0.94 (0.87, 0.97)	<0.001	0.97 (0.95, 0.99)	<0.001
Overall	0.97 (0.94, 0.98)	<0.001	0.92 (0.83, 0.96)	<0.001

* The average of the two raters was analyzed.

**Figure 2:**
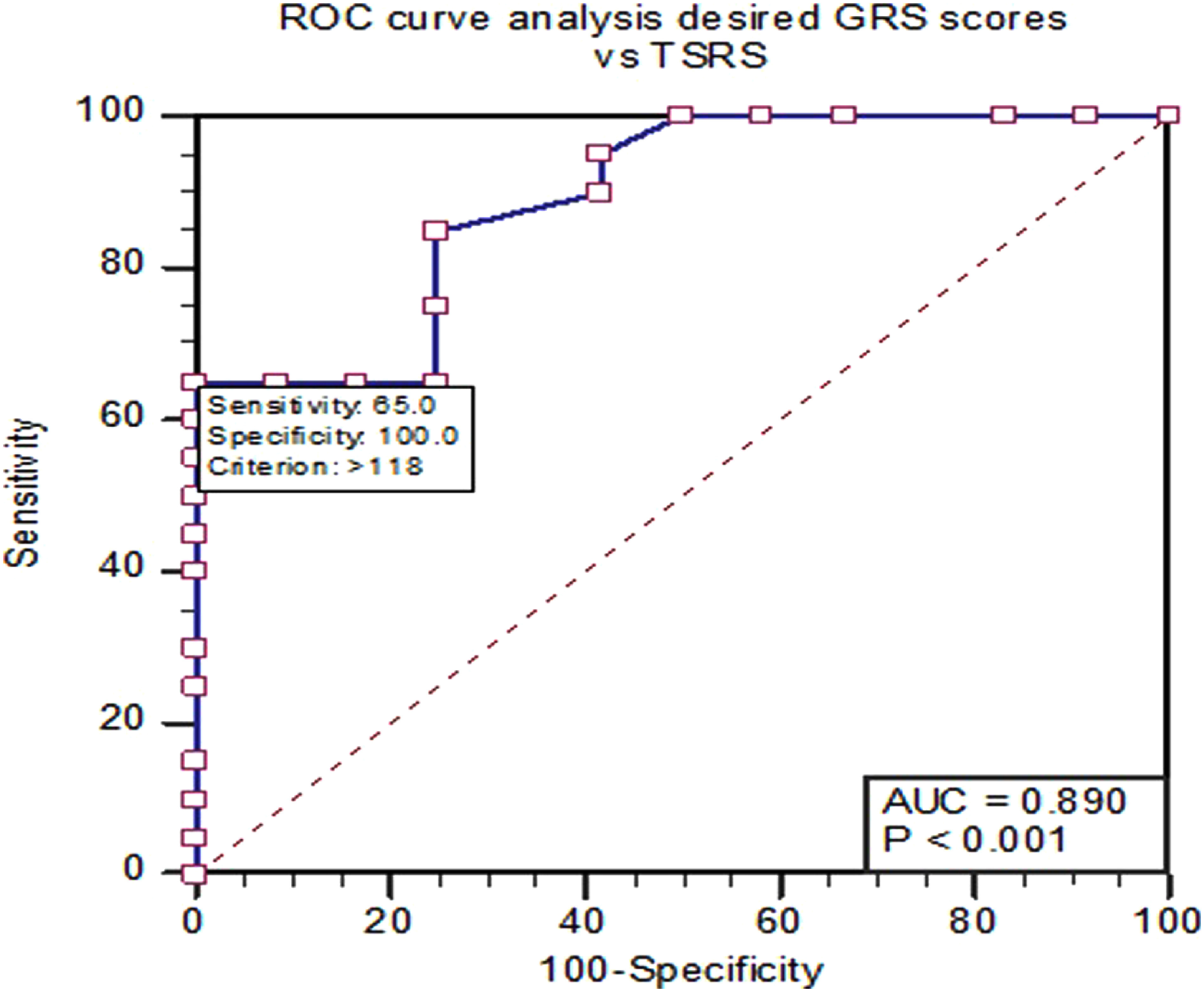
Fig. 2 Receiver operating characteristic (ROC) curve analysis showing the task-specific rating score cut points against 65% of the Global Rating Scale scores.

The TSRS cut point estimated on the ROC was 118, which corresponds to 65% of the overall GRS scores that can be used to discriminate participants who performed well from those who need further improvement (**Fig. 2**). Ninety percent of experts (n=9/10) obtained the desired 65% scores compared to the novices, only 50% of whom were declared successful (p=0.050).

Both the GRS and mean TSRS scores could discriminate the expert group from the novice group in overall scores (GRS: 27.8±3.13 and 22.16±6.24; p=0.011; and TSRS: 32.45±1.08 and 30.34±1.73; p=0.001). Instrument handling and the use of an assistant could not be differentiated by either the GRS or the TSRS. Like GRS, the TSRS also could not detect the difference between the groups in the “respect for tissue” and “time and motion” domains (**Table 3**). Experience in vascular surgery (months) and the number of embolectomies performed by experts were significantly higher than those in the novice group (95.4±58.0 vs. 3.64±3.89; and 97.0±47.8 vs. 2.64±47.8, respectively; p<0.001 for both groups).

**Table 3:** Table 3 Comparison of domain-specific means of GRS and TSRS scores among expert and novice groups, by task; n=32

Task	GRS^‡^			TSRS^†^		
	Expert n=10	Novice n=22	p-Value	Expert n=10	Novice n=22	p-Value
Respect for tissue	4.1±0.32	3.23±0.83	0.003	4.09±0.26	3.9±0.38	0.166
Time and motion	4.1±0.74	3.18±1.03	0.017	4.43±0.17	4.3±0.27	0.180
Instrument handling	3.6±0.7	3.36±1.19	0.565	4.76±0.32	4.63±0.18	0.144
Knotting and suturing	3.9±0.94	2.86±1.19	0.021	4.87±0.23	4.53±0.33	0.007
Use of assistant	3.7±0.63	2.95±1.22	0.081	4.6±0.57	4.25±0.7	0.178
Procedural flow	4.3±0.42	3.34±0.93	0.004	4.86±0.23	4.61±0.26	0.016
Quality of final product	4.1±0.57	3.23±1.04	0.019	4.85±0.23	4.12±0.57	0.001
Overall	27.8±3.13	22.16±6.24	0.011	32.45±1.08	30.34±1.73	0.001

^‡^ Global Rating Scale. ^†^ Average task-specific rating scale. * The p-value is derived from an independent t-test or the Mann–Whitney U test. Values are means±standard deviations.

## Discussion

This pilot study aimed to develop and validate a task-specific checklist to evaluate BAE procedures against GRS scores. To the best of our knowledge, this is the first study that has validated the TSRS for BAE for trainees. The overall TSRS was found to be a valid and reliable tool to evaluate the procedural steps of BAE. However, there was low reliability for some domains, such as time and motion and instrument handling. This warrants further modification of this pilot-tested TSRS checklist to improve its validity and reliability.

The GRS has been widely used to assess different surgical procedures, and despite being a validated tool, it has some inherent limitations of being generic and lacks specificity for various procedures.^2)^ This is one of the reasons that raised the need to develop procedure-specific evaluation checklists. There is a debate about the superiority of GRS over procedure-specific validated checklists. Ilgen et al.^12)^ collated the results of 45 studies in a meta-analysis and reported that the GRS is better able to differentiate expert performance and is more reliable than dichotomous checklists. However, the experts’ consensus is on using a combination of the GRS and validated procedure-specific checklists as the gold standard for procedure-specific assessments.^13, 14)^ The GRS overestimates respect for tissue and quality of products, whereas the TSRS does so for all other domains. We assumed a difference of <15% as an acceptable range; overall and all task-specific domains estimates fell within this range, except for knotting and suturing that exceeded this range. However, further modification is needed to make this difference even smaller for a robust assessment.

Our study demonstrated that both the GRS and TSRS can differentiate between expert and novice group performances. The differences in overall mean scores in both the groups were significantly different on both the checklists. This is intuitive as a higher mean score by the expert group could be explained by candidates having more experience in the field. Despite a significant difference in overall mean scores in both groups, the GRS and TSRS cannot differentiate instrument handling and use of assistant domains, whereas the TSRS cannot discriminate group differences in respect for tissue. This point may generate a hypothesis that this might be due to the inherent inability of these checklists to adequately assess these constructs. The TSRS, on the other hand, could not adequately differentiate the additional two domains of “respect for tissue” and “time and motion.” This can be further explained by the fact that the expert group either generally lacks expertise in these two domains or that this checklist needs further modification for a more robust evaluation in these domains.

This study demonstrated an acceptable validity and reliability of the TSRS; nonetheless, the question regarding its robust utility remains unanswered. Issues such as who will use this checklist under what circumstances and for what purposes raise questions about the utilization of this checklist. Similar issues with procedure-specific checklists have also been reported.^13)^ For intermediate-stake assessments, such as end-of-course tests, it is recommended that reliability coefficients should range between 0.80 and 0.89.^15)^ Our overall reliability coefficient for the TSRS fell in these ranges (r=0.85), thus validating the role of this checklist for its use could be restricted in intermediate-stake assessments. We propose that this TSRS should be used in workplace-based assessments, such as direct observations of procedural skills for general surgery and vascular surgery trainees. This checklist can also be used to evaluate an individual’s performance of the procedural steps in workshops or courses. High-stake examinations, such as national or international specialty exit examinations, should avoid using this rating scale. Besides the utility of the TSRS in assessment, this checklist can be a valuable tool and reference in the education and training of surgical trainees in simulation labs, wet labs, or operating rooms. Although the impact of utilizing this checklist, ultimately leading to improved outcomes in patients undergoing BAE, seems promising, no definite conclusion can be drawn at this moment because of the lack of available evidence.

Given the paucity of literature on such checklists and this being the first ever attempt to validate a task-specific checklist for BAE, we could not compare our results with other published evidence. Our results should be interpreted with caution because of the following limitations. There was only a small number of participants, especially in the expert group, and we could not assess stratified cut points. This is because of the evolving nature of vascular surgery as a speciality and because very few trained vascular surgery faculty were available for this study.^16)^ The findings of this study also showed the inability of the TSRS to adequately assess and differentiate some domains, such as instrument handling, use of assistant, and respect for tissue. Studies have shown that the use of nonbinary checklists or the Likert scale for assessments and reliability analyses using the ICC has inherent flaws,^17, 18)^ and this is applicable to our study as well. These limitations caution the use of this checklist in high-stake assessments. Further modification of this existing checklist to incorporate items in the abovementioned domains is certainly desirable.

## Conclusion

Overall, the TSRS was found to be a valid and reliable assessment tool for BAE; however, for some domains, such as instrument handling and time and motion, it has limited reliability, whereas for knotting and suturing, it showed a limited agreement between the two instruments. We could not prove the true educational impact of using the TSRS in acquiring expertise in embolectomy. Further work is warranted for the modification of this checklist by recruiting a larger number of participants from multiple centers.

## Supplementary Information

Appendices are available at the online article sites on J-STAGE and PMC.
