# Supplementary material for: Validation of Task-Specific Rating Scale for Open Balloon Catheter Arterial Embolectomy: An Assessor-Blinded Quasi-Experimental Pilot Study
Source: Ann Vasc Dis. 2022 Dec 25;15(4):289–94. doi: 10.3400/avd.oa.22-00047 (PMC9816037; doi:10.3400/avd.oa.22-00047)
Supplement: Supplementary Data [file avd-15-4-oa.22-00047-s001.pdf]

Appendix 1

GLOBAL RATING SCALE

Participant ID number: _____________ Date of assessment:____________

Time procedure started (min): _____________ Time procedure completed (min): _____________

Construct	1	2	3	4	5	TSRS corresponding items
**Respect for tissue**	Frequent unnecessary tissue force or damage to vessel		Careful tissue handling, occasional inadvertent damage		Consistently handled tissue carefully (appropriately), minimal tissue damage	**(1, 2, 14, 21)**
**Time and motion**	Many unnecessary moves		Efficient time & motion, some unnecessary moves		Clear economy of motion, and maximum efficiency	**(4. 5)**
**Instrument handling**	Repeated tentative or awkward moves, inappropriate use of instruments		Competent use of instruments, occasionally stiff or awkward		Fluid concise moves with appropriate instruments	**(3, 6–8)**
**Knotting and suturing**	Defective techniques resulting in poor tissue apposition and unsafe knots		Knotting and suturing usually reliable but sometimes awkward		Sound techniques and smooth action	**(16–19,22)**
**Use of assistant**	Consistently places assistant poorly or fails to use them		Appropriate use of assistant		Uses assistant to the best advantage at all times	**(20)**
**Procedural flow**	Frequently stopped and seems unsure of next move		Demonstrate some forward planning, reasonable progression		Effortless, obviously planned course	**(9–15)**
**Quality of final product**	Final product well below standard and likely to fail		Final product has deficiencies but would probably function adequately		Excellent final product with no flaws and likely to function well	**(23–26)**
**Total score:**						

_______________________ Assessor's signature

___________________ Assessor's Name

Appendix 2

TASK SPECIFIC RATING SCALE

Participant ID number: _________ **Date of assessment:_________________**

Time procedure started (min): _________ **Time procedure completed (min): _________**

**Construct**	**Not done (1)**	**Partially done (2)**	**Unsatisfactorily done(3)**	**Satisfactorily done (4)**	**Excellently done (5)**
**1. Control of blood vessels**<br>1. Use of appropriate clamps (bulldog, Fogarty, atraumatic)					
2. **Creation of transverse Arteriotomy**<br>2. Palpate artery before incision to ensure disease free site					
3. Appropriate blade (#11)					
4. Anterior wall, less than 1/3 circumference of artery					
5. Avoids trauma to posterior wall					
3. **Preparation of Balloon catheter**<br>6. Selects appropriate size of catheter					
7. Get air out of catheter by flushing & aspiration					
8. Checks balloon inflation & checks for leaks					
4. **Embolectomy**<br>9. Introduce the catheter hitting posterior wall					
10. Advances catheter proximal and then distal without excessive tension					
11. Withdraws with minimal tension					
12. Used forceps to retrieve thrombus over Arteriotomy					
13. Passes catheter first half way then full way					
14. Avoids excessive force & uses additional maneuvers as required					
15. The vessel is irrigated with the heparinized saline					

5. **Closure of Arteriotomy**<br>16. Selects appropriate suture (5/0 or 6/0 prolene)					
17. Selects appropriate vascular needle driver & forceps					
18. Suture placed outside-in (proximal artery), & inside-out (distal)					
19. Bites consistently spaced (2-3 mm apart)					
20. Instructs assistant to follow appropriate tension & direction					
21. Avoids excessive trauma/ handling the tissue with instruments					
22. Guides suture down to desired position					
23. Flushes prior to last few bites					
24. Ties the final suture with appropriate tension-no air knots					
25. Maximum of 8 throws on final knot					
26. Quality of final product					

________________________ ____________________

Assessor's signature **Assessor's Name**
